# Reducing the primate pet trade: Actions for primatologists

**DOI:** 10.1002/ajp.23079

**Published:** 2019-12-26

**Authors:** Marilyn A. Norconk, Sylvia Atsalis, Gregg Tully, Ana Maria Santillán, Siân Waters, Cheryl D. Knott, Stephen R. Ross, Sam Shanee, Daniel Stiles

**Affiliations:** ^1^ Department of Anthropology Kent State University Kent Ohio; ^2^ Professional Development for Good Chicago Illinois; ^3^ Pan African Sanctuary Alliance (PASA) Portland Oregon; ^4^ Departamento de Etología Instituto Nacional de Psiquiatría Ramón de la Fuente Muñiz Mexico City Mexico; ^5^ Centro Mexicano de Rehabilitación de Primates A.C. Vera Cruz Mexico; ^6^ Department of Anthropology Durham University Durham UK; ^7^ Barbary Macaque Awareness & Conservation Morocco; ^8^ Departments of Anthropology and Biology Boston University Boston Massachusetts; ^9^ Gunung Palung Orangutan Conservation Program West Kalimantan Indonesia; ^10^ Lester E. Fisher Center for the Study and Conservation of Apes, Lincoln Park Zoo Chicago Illinois; ^11^ Neotropical Primate Conservation Cornwall UK; ^12^ SOS Wildlife, Diani Beach Kenya

**Keywords:** collaborative research, commercialization of primates, illegal trafficking in live animals, social media

## Abstract

This commentary emerged from a panel presentation at the International Primatological Society Congress in Nairobi, Kenya, 2018. The goal was to provide regional updates on the status of primate removal from habitat countries, especially for the pet trade, and develop guidelines that could help primatologists address this critical problem. The trade in live primates includes those used as pets, in entertainment, and as subjects of biomedical experimentation, but here we focus on those primates destined for the pet trade. Such transactions are a hugely lucrative business, impacting hundreds of thousands of individuals annually and affecting the survival of wild populations. Being intimately familiar with primate social behavior, life history and biology, primatologists, whether they work with captive or wild primates, are in a unique position to understand the nature of the trade and attempt to counter its effects. In addition to updating the status of the primate pet trade, we provide recommendations that may help primatologists formulate a plan to deal, locally and regionally, with illegal trafficking in live primates. General guidelines include increasing awareness of local customs, policies and laws; developing collaborative research opportunities for local people; engaging in training/informational opportunities; and instructing on how to take action when encountering illegally‐trafficked primates.

## Research Highlights

- The primate pet trade continues to grow, impacting the survival of individuals and wild populations globally.
- Primatologists are in a unique position to reduce the number of primates in the pet trade stream by virtue of their knowledge of primate biology and behavior, and long‐term studies in habitat countries.
- Actions we can take include increasing awareness of local customs and laws, increasing scientific knowledge and designing collaborative research to empower local people, and avoid posing with primates in social media posts.

## Graphical abstract

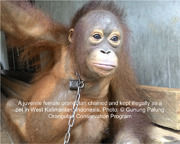

## 1 INTRODUCTION

The live primate trade consists of animals that are captured and removed from their native habitat and enter a local, national or international market for any reason (pets, entertainment, biomedical and pharmaceutical industries). This commentary focuses on the primate pet trade. It originated with a panel organized by S. Atsalis, M. Norconk and G. Tully for the 27th Congress (2018) of the International Primatological Society in Nairobi, Kenya. Panelists included concerned primatologists with personal or research experience on the pet trade. We seek to help primatologists, in particular those conducting field research, to develop plans to counteract the illegal trade in wild primates by reviewing relevant background information, and providing suggestions for how field and captive primate researchers can become more involved in reducing the entry of primates into the pet trade stream.

## 2 BACKGROUND: THE LIVE PRIMATE TRADE

The documented trade in live primates is lucrative and complex involving the capture and movement of hundreds of thousands of individuals per year for the biomedical, entertainment industry, and personal pet trade markets (Nijman, Nekaris, Donati, Bruford, & Fa, 2011). Using data from the Convention on International Trade on Endangered Species of Wild Flora and Fauna (CITES), Nijman et al. (2011) reported that the number of primates in trade increased steadily from 1995 to 2008. In 2015, primate trade volume was estimated to be $138M, an increase from $98M in 2012 (Observatory of Economic Complexity (OEC), https://atlas.media.mit.edu). Since 2008, China, Cambodia, and Vietnam have been the three largest exporters of live primates (https://atlas.media.mit.edu/en/profile/hs07/010611/#Exporters; UN Comtrade Database: https://comtrade.un.org/). These sites do not provide specific information on the destination of the primates or their source, although Nijman et al. (2011) found that the number of captive‐bred primates exported from CITES signatories from 1995 to 2009 exceeded the number of wild‐caught primates. They also suggested that the total number of wild‐caught primates were under‐reported for some years. Bush, Baker, and MacDonald (2014) found that all reports of CITES‐listed animals exported in the exotic pet trade were reported as “captive bred” on export.

As an example of the lucrative nature of documented (presumed legal) trade in live primates, exports from China in 2017 were valued at $48.1M, followed by Vietnam at $12M, and Cambodia at $11.3M. In the same year, 71% of China's exported primates were imported into the United States and 43% of Vietnam's and 55% of Cambodia's exports, respectively, were imported into Japan. The United States has remained the largest importer of live primates since 2009. The second‐largest importer during that period shifted from France to Qatar in 2015 and 2016.

The volume of the undocumented (illegal) trade is estimated to be even higher than the documented trade, although no realistic estimate of the actual number of animals exists (Estrada et al., 2018; Reuter & Schaefer, 2017; Rosen & Smith, 2010; Stiles, Redmond, Cress, Nellemann, & Formo, 2013). The accuracy gap is due to accountability issues, inadequate enforcement of existing regulations, inaccurate or incomplete population assessments, and secrecy. Some countries (e.g., Indonesia—Shepard, 2010; Suriname—Ouboter, 2001; Vietnam and China—Bush et al., 2014; Yiming & Dianmo, 1998) continue to export Appendix 1 species (those considered most endangered by CITES) despite having indicated acceptance of the import/export terms in CITES. But, poor enforcement of existing regulations is a ubiquitous problem (Bergin, Atoussi, & Waters, 2018; Freund, Rahman, & Knott, 2016; Maldonado, Nijman, & Bearder, 2009; Nijman, 2017; Shanee, 2012, 2017). CITES has little enforcement capability and must rely on law enforcement agencies and reporting accuracy in primate range countries (Márquez‐Arias, Santillán‐Doherty, & Arenas‐Rosas, 2017; Wyler & Sheikh, 2008). And, it may be difficult to verify assessments of population growth or decline, criteria that are routinely used to designate export quotas (Challender, Harrop, & MacMillan, 2015; Meijaard, Wich, Ancrenaz, & Marshall, 2012; Plumptre, Sterling, & Buckland, 2013). Finally, efforts to limit the transfer of primates across international boundaries may be overwhelmed by demand for some species for pets or for biomedical research such as *Nycticebus* spp. (lorises), *Saimiri* spp. (squirrel monkeys), *Cebuella pygmaea* (pygmy marmosets), *Aotus* spp. (night monkeys) and several species of lemurs (e.g., Fuller, Eggen, Wirdateti, & Nekaris, 2018; Maldonado, 2018; Musing, Suzuki, & Nekaris, 2015; Nijman, Spaan, Rode‐Margono, & Nekaris, 2017; Simoes & Hidalgo, 2011; Svensson et al., 2016).

Nevertheless, efforts are being made on many levels to reduce the entry of individuals into the live primate trade stream. These efforts include education of local people and others who reside in the same habitats as primates (Freund et al., 2019); community development projects that provide alternative livelihoods to hunting and wildlife trafficking (Challender et al., 2015; Horwich et al., 2010); creation of new protected areas (PAs) in habitats of high biodiversity and strengthening protection of existing PAs (Le Saout et al., 2013); funding for forest rangers whose presence and efforts deter poachers; and both stronger legislation and consistent enforcement of existing legislation (Meijaard et al., 2012; Phelps, Biggs, & Webb, 2016). Additionally, we recognize increasing corporate responsiveness to the commercial transportation of live primates in response to public pressure. For example, several airlines now refuse to transport primates internationally (Grimm, 2018).

## 3 SUPPLY AND DEMAND FOR PET PRIMATES

**Figure 1:**
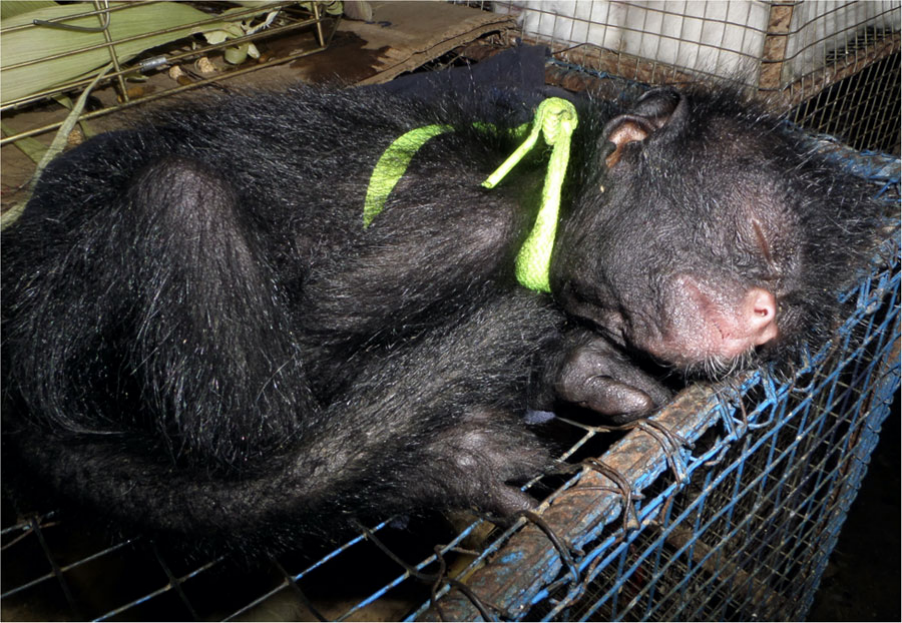
Spider monkey, *Ateles chamek*, for sale in Bellavista market, Pucallpa, Peru. Photo credit: Noga Shanee (Neotropical Primate Conservation)

**Figure 2:**
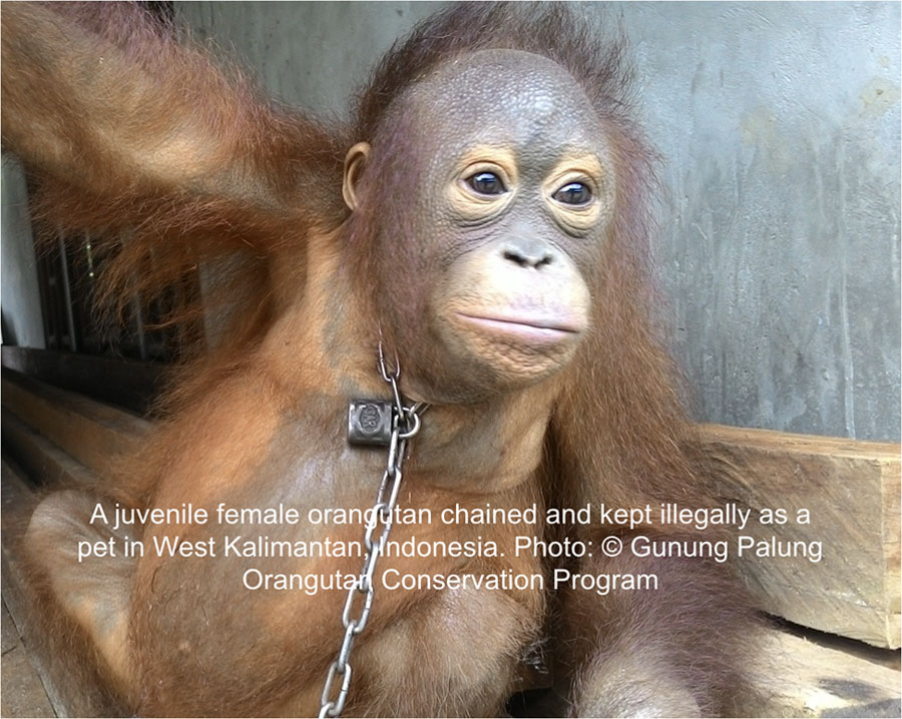
A juvenile female orangutan chained and kept illegally as a pet in West Kalimantan, Indonesia. Photo: © Gunung Palung Orangutan Conservation Program with an example of an embedded caption

Primates can be attractive as pets because they are viewed as cute and ‘funny’ and often behave in familiar ways that are similar to our own behavior (Estrada et al., 2017; Marshall & Wich, 2016a; Phillips et al., 2014). Research has demonstrated that their attractiveness is influenced by how they are portrayed in popular media such as television, movies and commercial advertisements (Aldrich, 2018; Ross, Vreeman, & Lonsdorf, 2011). While in some countries pet primates have been purposefully bred in captive colonies, they may also enter the pet trade from the wild both intentionally, via wildlife traffickers (Freund et al., 2016; Nijman, 2017; Phelps et al., 2016; Shanee, 2012; Stiles, 2016; Stiles et al., 2013; van Uhm, 2016; Figures 1 and 2) and incidentally, when local hunters kill females with infants that are then kept as pets or sold (Beck, 2010; Stiles et al., 2013). Furthermore, there is concern that wildlife crime—the 4th largest type of international crime—may put animals that inhabit PAs at risk, particularly great apes (Dudley, Stolton, & Elliott, 2012). As PAs increasingly serve as “safe harbors” for relatively high densities of animals, they may become hotspots for hunters and traffickers who often operate with impunity (Nijman, 2017).

There is also concern that the traditional opportunistic capture of animals for the pet trade (as in this case of orangutan infants) has shifted to more organized trafficking activities Freund et al. (2016). For example, in Kalimantan and Sumatra, Indonesia, there are two known ape traffickers with holding grounds based in the Jakarta area, and two others in central and east Java, that acquire primates from local collectors and sell them on social media or to contacts they have in zoos and safari parks (D. Stiles, personal communication, November 25, 2019). In Mexico, organized wildlife crime groups take advantage of the same trafficking routes used in the drug trade (Alvarado‐Martínez, 2012). A similar problem is gaining traction in Peru (Shanee, 2012) where wildlife trafficking is compounded by poor enforcement of national laws and international conventions (Shanee, Mendoza, & Shanee, 2017).

Shanee (2012) noted that local politicians in Peru were influential in contributing to or reducing the local pet trade. In Amazonas state, lax interpretation of wildlife laws failed to deter politicians and their families from assembling their own menageries of exotic animals while in neighboring San Martin state, stricter interpretation and application of the same law resulted in the rescue of many animals from commercial establishments (S. Shanee, in preparation). In both cases, the decisions made by local leaders affected the incidence of local wildlife crime.

**Figure 3:**
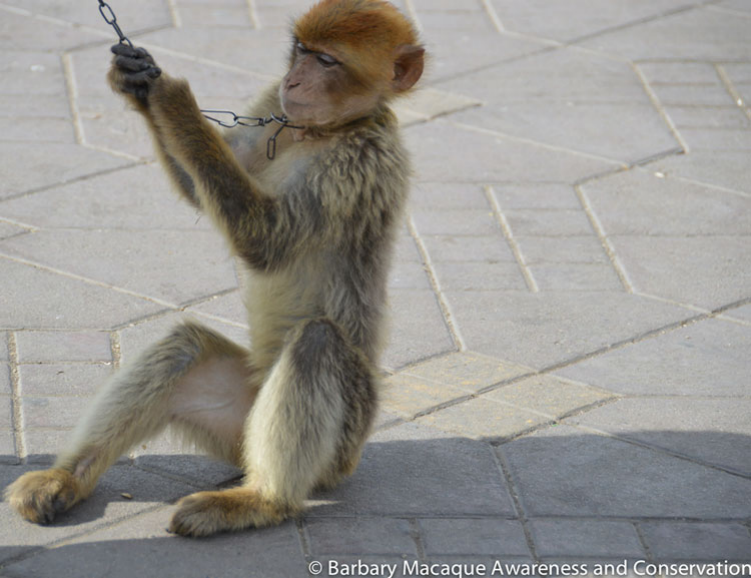
Barbary macaque used as a photo prop at a market in Marrakech, Morocco. Photo: © Barbary Macaque Awareness and Conservation

As a species, barbary macaques have been greatly affected by both internal and external illegal trade in Morocco. van Uhm (2016) estimated that ~200 macaques were smuggled into the European Union annually. Despite the species being reclassified on CITES Appendix I in 2016, trade does not appear to have diminished and the authorities have failed to consistently enforce the law against the open sale and use of Barbary macaques as a photo prop for tourists. Jmaa El Fnaa square in Marrakech is a magnet for both local and international tourists. Given special permission by the authorities, solicitors (or touts) exploit macaques as photo props (Figure 3). The square is also where both national and international visitors initiate the purchase of wild‐caught infant macaques despite this activity being illegal (Bergin et al., 2018).

On the demand side, many cultures have historically maintained primates as pets for social‐status seeking (Márquez‐Arias et al., 2017; Shanee, 2012) and medicinal and spiritual reasons (e.g., Nekaris, Shepherd, Starr, & Nijman, 2010; Nijman & Nekaris, 2014; Reuter et al., 2018; Waters, Bell, & Setchell, 2018). Moreover, international demand is driven by greater access to wealth, advertising on the internet (Bergin et al., 2018) and commercialization in films and videos (Aldrich, 2018; Nekaris, Campbell, Coggins, Rode, & Nijman, 2013). The ease of financial exchange and speed of air transportation heavily impact the international trade of some species (e.g., pygmy marmosets, Mongabay 2016, https://news.mongabay.com/2016/02/the‐dangers‐of‐chinas‐thumb‐monkey‐trend/ and slow lorises, Nekaris et al., 2013).

## 4 INTERVENTIONS BY PRIMATOLOGISTS TO REDUCE THE PET TRADE—WHAT CAN WE DO?

Many primatologists are in a unique position to increase awareness of and/or reduce the national and international trade in wild primates. Field primatologists often develop close relationships with local people and are aware of primate habitat requirements, population demography, and social behavior. Most field primatologists, however, are not trained to take action when confronted with primate trafficking. Given this gap in knowledge and limited formal direction from our professional associations (International Primatological Society policy statement, “Trade in Primates Captured in the Wild”), we provide a summary below of recommendations generated as a result of the panel presentations at the IPS 2018 Congress in Nairobi, Kenya. General guidelines for longer‐term preparation are followed by suggestions for immediate action when confronted with illegal primate pet trade situations during the course of travelling or conducting research. All primatologists, whether they do field research or not, should be aware that their study subjects are at risk of trafficking.

### 4.1 General guidelines/Long‐term planning

#### 4.1.1 Increase your knowledge of local customs, history and laws as they pertain to wildlife trafficking

Among the most useful actions researchers can take is to identify, and be prepared to contact, specialized local enforcement agencies, rescue centers and Nongovernmental Organizations (NGOs) working in areas where wildlife trafficking occurs. Primatologists should make themselves aware of these resources even before witnessing any activity so as to be prepared to act quickly when necessary. In many cases local authorities may be apathetic or reluctant to act because of a lack of funding or expertise, that is, general reluctance to confiscate illegally held trafficked animals. In such cases, regular follow‐up calls may encourage authorities to act or one may need to contact more than one enforcement entity.

Similarly, highlighting positive actions of local authorities in local media or online may reinforce their decisions. It is common for local authorities to feel that cases involving just one animal kept as a pet are not serious crimes. The incident may be ignored, or, if a confiscation is made, no punishment may result. Both in‐country and international primatologists should proceed with caution in such cases to avoid being perceived as interfering and possibly making future collaboration with local agencies challenging. Contacting NGOs with social media presence can encourage the authorities to act to avoid public criticism nationally (Waters, personal observation). Primatologists may also be able to coordinate capacity‐building training sessions for local authorities by working with knowledgeable local groups.

Primatologists should also be aware of emerging changes in the local social and business environments, particularly the influx of new commercial enterprises (e.g., oil palm plantations, logging, land trafficking, ranching, and mining) that could impact habitat loss and lead to an increase in primate trafficking (e.g., Freund et al., 2016; Shanee & Shanee, 2016).

#### 4.1.2 Develop personal relationships with local people

Developing personal relationships (from local residents, to field assistants and local leaders) may be the most promising approach to deterring the illegal pet trade at its source. Primatologists should strive to create social networks that include community members and local authorities to promote truthful information about local primates and other animals targeted for the illegal pet trade and their importance in shared ecosystems with humans.

Deep, meaningful engagement and trust‐building between local people and primatologists in Morocco led to conservationists and forest users, such as shepherds, sharing information about Barbary macaques. The conservationists used this opportunity to link the forest users and their home with the Barbary macaque's unique status as the only North African primate. These strategies made some men view the animals differently and develop a sense of pride in the species, protecting it from poachers and other threats (Waters et al., 2018).

#### 4.1.3 Increase the scientific capacity of local people

Increasing scientific capacity through workshops for local people, as well as structured training for students that intend to become professionals, are perhaps the most effective ways to affect primate conservation (Meijaard et al., 2012). In many countries, foreign researchers are required to support local students. Primatologists working outside of their home countries should embrace these opportunities to help train the next generation of in‐country scientists whose voices could have a much greater impact on policies within their country.

Moreover, community workshops have been shown to lead to collaborative approaches that reduce hunting and capture of endangered animals (Horwich et al., 2010; Shanee, 2012; Shanee & Shanee, 2015). Primatologists who are also educators should volunteer to give compelling, well‐informed lectures about their research and local conservation to targeted audiences: residents, companies, governmental representatives and policy makers, schools and universities, children and adults. A survey done in Mexico showed that even in range countries, especially in big cities, residents are unaware of the importance and even the presence of primates in the country (Márquez‐Arias, Arenas‐Rosas, & Santillán‐Doherty, 2013).

It is also essential for primatologists to help park administrators recognize the importance of integrating local people into the protection and maintenance of protected areas. In our experience, there are many examples where communities have lived next to parks for many years but have never visited them. Designing programs that introduce school children to parks are an effective way to change future attitudes (Freund et al., 2019), but we should also recognize the importance of long‐term investment in repeated education programs (workshops, trail walks, lectures, and so on) for maximum effectiveness.

**Figure 4:**
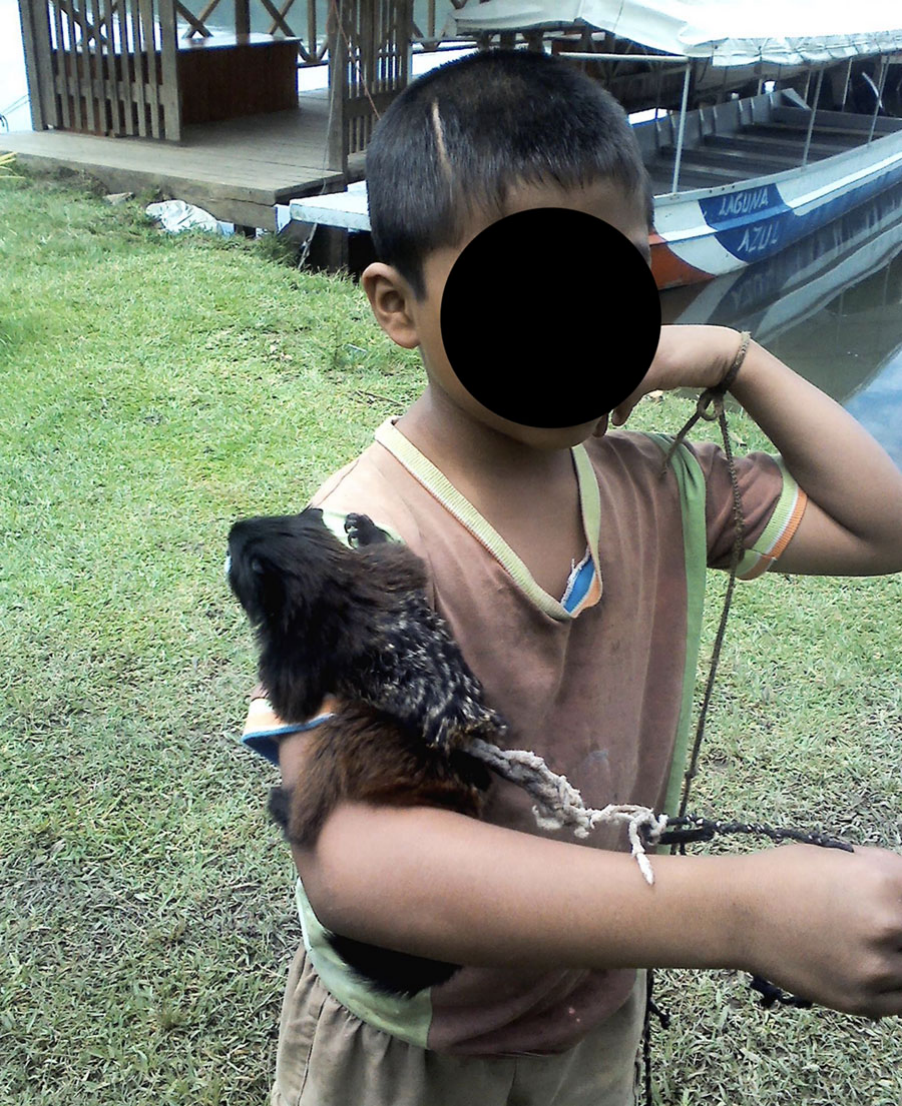
Tamarin offered for use as a photo prop, Sauce, Peru. Photo credit: Sam Shanee (Neotropical Primate Conservation)

**Figure 5:**
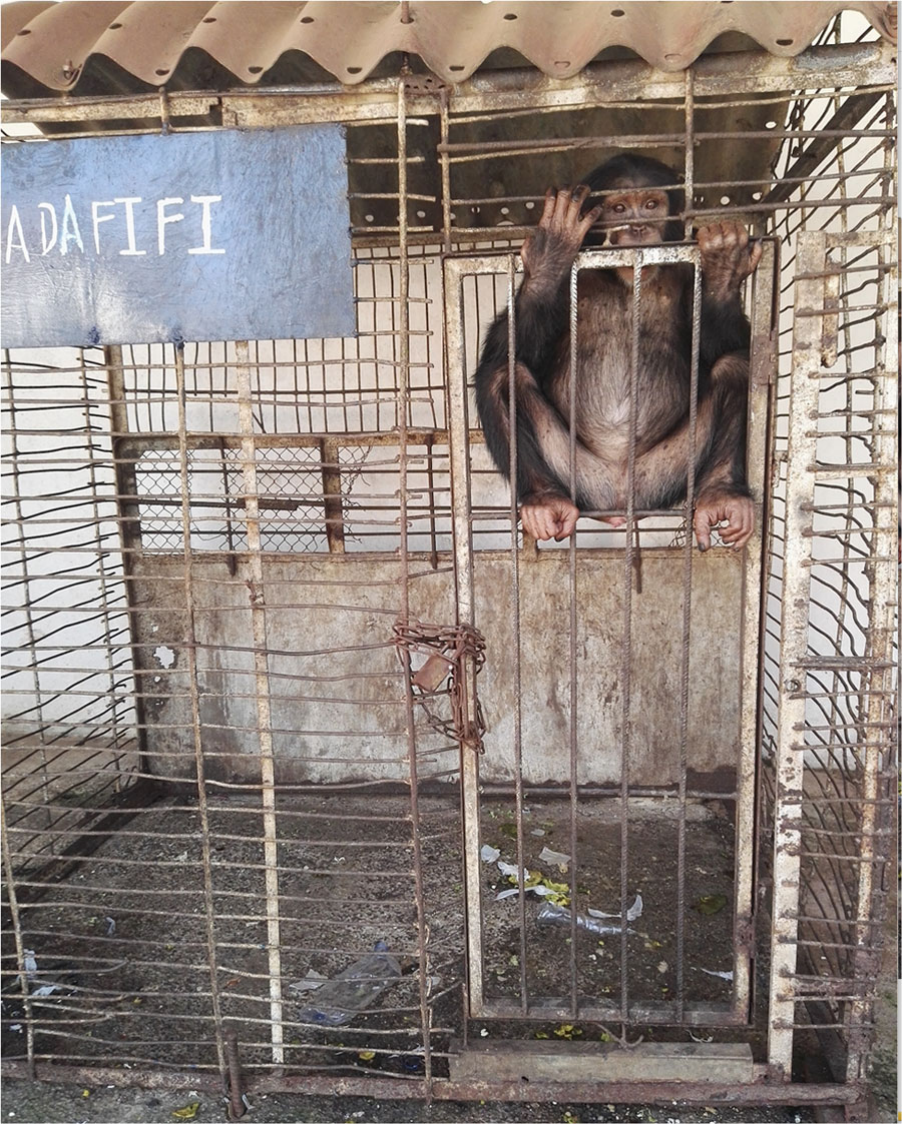
Fifi, a 10‐year‐old western chimpanzee, has spent almost her entire life in a small cage in the parking lot of a hotel in Guinea‐Bissau, West Africa. Photo credit: Marie Laforge

Collectively, we can take advantage of a global increase in nature tourism by engaging with local NGOs or governmental organizations to provide instructional materials for hotels, tourist sites, and national parks. Shanee (2012) found that live primates and other wildlife were used as “traditional jungle décor” in tourist sites in the Peruvian Amazon (Figure 4). But, tourist hotspots (e.g., hotels, Figure 5) may also be exemplary locations for primatologists to educate both local tour operators and tourists themselves by becoming active participants and providing help with educational programs and signage.

#### 4.1.4 Design collaborative research

Meijaard et al. (2012) encouraged the development of collaborative projects that integrate experts in related fields and provide large‐scale systematic information on primate population size, density and habitat requirements. Collaborative research may facilitate community involvement and provide insight into cultural perspectives on the pet trade (Nekaris et al., 2010; Reuter & Schaefer, 2017; Reuter et al., 2018; Waters et al., 2018). If the possession of exotic pets is embedded in traditional cultural attitudes, then understanding the source of the tradition may be useful in providing explanations to deter it. It will also be beneficial to think outside the traditional boundaries of primate research and become familiar with local law, economics, social and cultural traditions, forestry and other relevant fields in which people working on similar issues from different perspectives can provide more comprehensive solutions (Blair, Le, & Sterling, 2017; Marshall & Wich, 2016b; Meijaard et al., 2012; Waters & El‐Harrad, 2013).

Primatologists should strengthen connections with local people and provide specific information on conservation planning and practical implementation of antipoaching efforts in both regional reports and local publications. We also encourage primatologists to disseminate information in scientific and popular journals or newspapers, particularly in local languages, to publicize the plight of animals captured for the pet trade (e.g., Fuller et al., 2018).

#### 4.1.5 Collaborate with professional organizations

The International Primatological Society webpage (internationalprimatologicalsociety.org) lists 24 affiliated regional and country‐based primate societies. Our focus in this commentary is on what we, as primatologists, can do to reduce the number of primates entering the pet trade. The number of wild‐caught animals in the pet trade is difficult to calculate (Bush et al., 2014; Shanee et al., 2017), but it is increasing by most estimates (e.g., Nijman et al., 2011). Primates tend to enter the pet‐trade stream by being taken from wild populations. Many of the IPS affiliates are grass‐roots organizations that are centrally positioned to provide cultural‐ and language‐specific information to local governance officials, community leaders, wildlife police, and local people, and also may be able to help document the local primate pet trade. Organizations originating in non‐habitat countries (e.g., American Society of Primatologists, Primate Society of Great Britain, European Federation for Primatology, and the International Primatological Society) should provide financial and collegial support for regional organizations, and contribute to collaborative research and conservation initiatives as part of their research programs.

#### 4.1.6 Set social media guidelines

Social media platforms (e.g., Facebook, Twitter, and YouTube), as well as popular films that show primates as pets or wearing human clothes, have enhanced the attraction and facilitated the acquisition of wild animals as pets. Nijman and Nekaris (2017) found that the number of species of owls, both common and rare, increased in local markets in Indonesia following the release of Harry Potter films. The demand for otters as pets increased with the number of listings on social media in Thailand (Siriwat & Nijman, 2018). A survey of social media posts originating in Africa that contributed to the (illegal) international trade of gray parrots cited social media as a variable in the persistent high level of trade (Martin, Senni, & D'Cruze, 2018). We also want to emphasize that large‐scale surveys have demonstrated that photographs that show even minimal human contact (i.e., humans touching nonhuman primates or simply sharing space with them) may increase the likelihood that primates will be considered to be suitable pets (Leighty et al., 2015; Ross et al., 2011).

**Figure 6:**
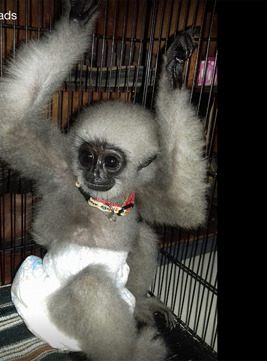
Infant gibbon offered for sale on Instagram in Indonesia

Ironically, images of humans caring for infant primates can have negative consequences for wild populations (e.g., Musing et al., 2015; Nekaris et al., 2013; Stiles et al., 2013). Images of care given to primates in rehabilitation centers may feed the view that “baby primates make cute pets” and exacerbate the pet trade problem (Figure 6).

If photos of humans with primates must be shared, then an appropriate caption explaining the context should be included. Still, the danger is that the contextual information will not be shared with the photo. We recommend that the information be embedded in the photo itself similar to an infographic or informative watermark (see Figure 2). Such amendments will enable photos to be traced more easily and avoid sharing them out of context. People and NGOs working closely with primates should err on the side of caution and exclude photos of people and primates together from their social media pages and websites.

Given its uniquely broad reach, social media provides tools to educate and inform, but primates are increasingly being sold on Facebook and Instagram contrary to their Terms of Service. Unfortunately, these online platforms have refused to cooperate with law enforcement citing privacy concerns (Stiles, 2019). Still, we can engage in judicious and intentional use of electronic media that includes accurate information such as the common name of the species, general photo location, behavior, and conservation status (Nekaris et al., 2013; Waters & El‐Harrad, 2013) while avoiding specific information regarding location (e.g., excluding GPS coordinates from smart phone photos).

### 4.2 Taking action

We are aware that primates are valuable commodities. Interrupting sales transactions and movements, or confronting sellers on streets or in markets may come with personal risks. Therefore, we contributed to this commentary with the belief that all field primatologists should become more aware of cultural, economic, commercial, legal, and educational impacts on the live primate trade in communities where we live and work, and to encourage primatologists to consider how they could become a counterforce to the primate pet trade.

#### 4.2.1 Do not purchase the animal

Buying wildlife from dealers or traffickers, or otherwise providing them with compensation, will give them resources to acquire more wild animals, exacerbating the trade. We should understand that purchasing animals will reinforce the behavior.

#### 4.2.2 Do not act alone

Contact local people you trust for advice about relevant wildlife protection authorities. Primatologists who find evidence of wildlife trafficking are encouraged to communicate relevant information about the location, species, and context of the sale with local law enforcement agencies or NGOs involved in wildlife rescue. Most primate range countries offer an anonymous reporting service through phone call or internet. If an organization or agency is positioned to take action, they are likely to have more experience with safely managing interactions with traffickers and will be more familiar with the laws of the country. Ideally, the traffickers will be arrested and/or fined in addition to the animals being confiscated.

#### 4.2.3 Do follow up

Calling, writing to or visiting local wildlife authorities increases opportunities to engage with local enforcement agencies and helps reinforce your interest in and support of their services.

#### 4.2.4 Do support good actions

Positive publicity for local wildlife authorities that may have insufficient resources for monitoring and tracking illegal trade could make a difference the next time they are called upon to take action. Such action may include communication via social media sites or writing editorials in local newspapers or magazines calling attention to the danger of trafficking to primate well being.

## 5 THERE MAY BE UNINTENDED CONSEQUENCES

We cannot offer a prescription for success, only guidelines for intentional action to deter the primate pet trade. It is critical that primatologists are involved at the ground level; that they are aware, involved, and support strategies and policies to reduce the illegal trafficking of nonhuman primates. We believe that our long‐term presence at field sites, appreciation for cultural patterns, and interest in gathering and exchanging information can provide local incentives to avoid the illegal capture of wild primates. Our input at this stage is critical. Post capture, we may have less control. For example, the Moroccan NGO, Barbary Macaque Awareness & Conservation (BMAC), found that confiscating photo‐prop macaques and fining their owners did not prevent the touts from re‐offending. The fines were rarely paid and the touts continued their trade without serious penalty—although they did lose their capital asset—the macaque. Thus, enforcing Moroccan wildlife protection laws by confiscating Barbary macaques resulted in the negative and unforeseen effect of increasing the demand for wild‐caught infant macaques for the photo‐prop trade. BMAC's experience demonstrates the difficulties that conservationists may experience whilst trying to enforce laws in cultures where the legal infrastructure is procedurally slow.

What is lacking in many cases is support for existing—and establishing new—rescue centers. The paucity of safe locations to place confiscated animals is an unfortunate obstacle when trying to motivate authorities to act. Knowing that an animal will probably be euthanized is disheartening to authorities and the public, disincentivizing a willingness to seize pet primates. Most primatologists are not in a position to set up a rescue center, but they can certainly find ways to provide much‐needed support for existing ones.

## 6 CONCLUSION

Primatologists have the tools and are in a position to effect change in the pet trade locally and internationally. We are educators and researchers; we interact closely with local people; we know the behavior, ecology, and life history strategies of the primates we study; and we often have long‐term investments in the countries in which we work or live. Many of us have seen primates for sale on street corners and markets or kept as pets in back‐yard cages in habitat countries or in pet stores and circuses in North America. Doing our homework to understand the attitudes of local people and the cultural underpinnings of how primates enter and move through the pet trade are critical to developing solutions to deter it. We encourage all primatologists to generate a personal plan of action tailored to local circumstances, avoid circulating pictures of themselves with primates, and engage with local organizations or individuals who can effectively intervene to deter the primate pet trade.

## AUTHOR CONTRIBUTIONS

We clarify the participation in this piece by noting that S. Atsalis, C. Knott, M. Norconk, S. Ross, A. M. Santillán‐Doherty, G. Tully, and S. Waters attended and participated in the panel at IPS‐Nairobi, 2018. D. Stiles and S. Shanee agreed to participate, but were unable to attend the meeting. They contributed data and comments that were included in the panel and subsequently contributed to writing the commentary.

## ETHICS STATEMENT

The authors declare that their research adhered to the joint International Primatological Society and American Society of Primatologists Code of Best Practices for Field Primatology, 2014.
